# Collaborative dynamics and shared motivation: exploring tobacco control policy development in Zambia

**DOI:** 10.1093/heapol/czae042

**Published:** 2024-11-18

**Authors:** Adam Silumbwe, Miguel San Sebastian, Joseph Mumba Zulu, Charles Michelo, Klara Johansson

**Affiliations:** Department of Health Policy and Management, School of Public Health, University of Zambia, PO Box 50110, Lusaka, Zambia; Department of Epidemiology and Global Health, Umeå University, Umeå 901 87, Sweden; Department of Epidemiology and Global Health, Umeå University, Umeå 901 87, Sweden; Department of Health Policy and Management, School of Public Health, University of Zambia, PO Box 50110, Lusaka, Zambia; Department of Epidemiology and Biostatistics, School of Public Health, University of Zambia, PO Box 50110, Lusaka, Zambia; Strategic Centre for Health Systems Metrics (SCHEME), Global Health Institute, Nkwazi Research University, PO Box 50650, Lusaka, Zambia; Department of Epidemiology and Global Health, Umeå University, Umeå 901 87, Sweden

**Keywords:** Tobacco policy, collaboration, shared motivation, trust, legitimacy, commitment, Zambia

## Abstract

In Zambia, efforts to produce a tobacco control policy have stalled for over a decade, and the country is not yet close to developing one. Limited studies have explored the dynamics in this policy process and how they affect the attainment of policy goals and outcomes. This study explored how collaborative dynamics within tobacco control policy development shaped shared motivation among stakeholders in Zambia. The study used a qualitative case study design that adopted a collaborative governance lens, comprising an in-depth exploration of the tobacco control policy working group meetings and their internal collaborative dynamics. The integrative framework for collaborative governance, which identifies mutual trust, mutual understanding, internal legitimacy and shared commitment as key elements of shared motivation, was adapted for this study. Data were collected from 27 key informants and analysed using thematic analysis. Several collaborative dynamics thwarted mutual trust among tobacco control stakeholders, including concerns about associated loyalties, fear of a ban on tobacco production, silo-mentality and lack of comprehensive dialogue. All stakeholders agreed that the limited sharing of information on tobacco control and the lack of reliable local evidence on the tobacco burden hindered mutual understanding. Diverse factors hampered internal legitimacy, including sector representatives’ lack of authority and the perceived lack of contextualization of the proposed policy content. Acknowledgement of the need for multisectoral action, lack of political will from other sectors and limited local allocation of funds to the process were some of the factors that shaped shared commitment. To accelerate the development of tobacco control policies in Zambia and elsewhere, policymakers must adopt strategies founded on shared motivation that deliberately create opportunities for open discourse and respectful interactions, promote a cultural shift towards collaborative information sharing and address unequal power relations to enable shaping of appropriate tobacco control actions in respective sectors.

Key messages

- Tobacco control policy development is a controversial issue, particularly in countries where tobacco has a significant economic value.
- In Zambia, efforts to develop a tobacco control policy have been ongoing for over a decade, but the country is not yet close to developing one. Building a shared motivation among tobacco control stakeholders is critical to overcome barriers and improve the effectiveness of collaboration. Shared motivation is even more important for health policy processes when there are conflicting interests.
- We note a limited sense of shared motivation among the tobacco control stakeholders in Zambia, shaped by collaborative dynamics that exacerbate a lack of trust, mutual understanding, internal legitimacy and shared commitment.
- To foster trust, it will be essential to adopt strategies that create opportunities for open discourse and respectful interactions. Improving mutual understanding will require promoting a cultural shift towards collaborative information sharing, while enhancing legitimacy and commitment requires tobacco control stakeholders to have the requisite power and authority to influence appropriate policy actions in their respective sectors.

## Introduction

Health policy-making is a highly contested process, involving multiple actors, with diverse interests, often influenced by a variety of contextual factors (Gilson *et al*., 2018). According to Sabatier and Weible (2019) power dynamics are the main consideration and stakeholders compete to promote their beliefs about policy problems and potentials solutions. Tobacco control policy process is a salient example of the innate complexities of health policy-making, particularly in low- and middle-income countries (LMICs) where the cultivation, production and sale of tobacco play a substantial role in their nation’s economic activities (World Health Organization, 2017). One of the main challenges is that in LMICs, governments often face the painstaking decision of trading off the economic benefits of tobacco farming/manufacturing vs protecting the health of their citizens (Kumar *et al*., 2022).

In Zambia, there are approximately 18, 000 tobacco farmers, contributing to the country’s annual earnings of ∼$140 million from tobacco leaf exports (Tobacco Board of Zambia, 2021). Efforts to develop a tobacco control policy have been ongoing for over a decade, but the country is not yet close to developing one, and current tax rates do not align with those proposed by the World Health Organization Framework Convention on Tobacco Control (WHO FCTC) (Labonté *et al*., 2018). Studies have documented several impediments (Lencucha *et al*., 2016; Ruckert *et al*., 2022), some of which are common to other settings (Gilmore *et al*., 2015; Ulucanlar *et al*., 2016; Matthes *et al*., 2021). For example, the growing presence of the tobacco industry in the local economy has crippled government attempts to regulate it (Appau *et al*., 2020). Like in other LMICs, the tobacco industry opposes the tobacco control policy, claiming that such a policy is detrimental to development and is driven by foreign agendas rather than national interests (Ruckert *et al*., 2022). Moreover, the fragmentation and strong opposition within government departments, some of which argue against supply-side measures of tobacco control, have also hindered tobacco control policy development (Lencucha *et al*., 2016).

In 2008, Zambia ratified the WHO FCTC, which establishes a legal framework for the adoption of comprehensive tobacco control measures (World Health Organization, 2015). Since 2009, Zambia has engaged several stakeholders through consultative meetings led by the Ministry of Health (MoH) to develop a tobacco control policy (Ruckert *et al*., 2022). Government sectors, including Agriculture, Trade and Commerce, Local Government and Housing, Justice, and Community Development and Social Welfare, civil society organizations (CSOs), such as the Centre for Policy and Trade, the Non-communicable Diseases Alliance and the Tobacco Free Association of Zambia, and the WHO have actively participated in the meetings. To date, these meetings have yielded two draft tobacco control policies, one in 2010 and another in 2020. Both drafts seek to promote smoke-free environments, as well as regulate tobacco advertising, promotion, sponsorship, labelling and sales (Zambia National Parliament, 2020). However, the cabinet, the body of government ministers responsible for endorsing legislation to be considered by parliament, did not approve both draft policies (Labonté *et al*., 2018). On both occasions, the cabinet demanded further consultations due to the lack of consensus among involved stakeholders (Tobacco, 2021).

Such consultative meetings require skilful facilitation to bring together policy stakeholders with differing views and interests (De Leeuw, 2017). However, our recent study showed that the engagement in these meetings has been hampered by adverse legal and socio-economic environments in which the policy process evolves; poor planning of meetings and frequent changes in tobacco focal point persons; lack of active and meaningful participation; and communication challenges among the stakeholders (Silumbwe *et al*., 2023). As such, building a shared motivation among the tobacco control stakeholders becomes critical to addressing these challenges (Emerson and Nabatchi, 2015a). Shared motivation can help overcome barriers and improve the effectiveness of collaboration among stakeholders in the policy process, leading to the attainment of policy goals and outcomes (Oh and Bush, 2016).

Despite the importance of shared motivation in facilitating collaboration, to the best of our knowledge, there is a paucity of studies that describe the specific role it plays in policy processes, including how and why failure to attain it may affect policy goals and outcomes. Using the case of the tobacco control policy in Zambia, our study aimed to explore how the collaborative dynamics within the policy process shaped shared motivation among stakeholders involved in tobacco control policy-making. This study could help understand the process of health policy-making in general, especially where there are conflicting interests and for countries with a similar context to Zambia.

## Materials and methods

### Study design

This was a qualitative case study that adopted the integrative framework for collaborative governance to explore the tobacco control policy development in Zambia (Baxter and Jack, 2008; Emerson *et al*., 2012). The case study drew on interviews with stakeholders who have been participating in the consultative meetings to develop the tobacco control policy. It explored the inherent collaborative dynamics and the socio-political context in which these consultative meetings have occurred (Baskarada, 2014).

#### Integrative framework for collaborative governance

**Figure 1.**
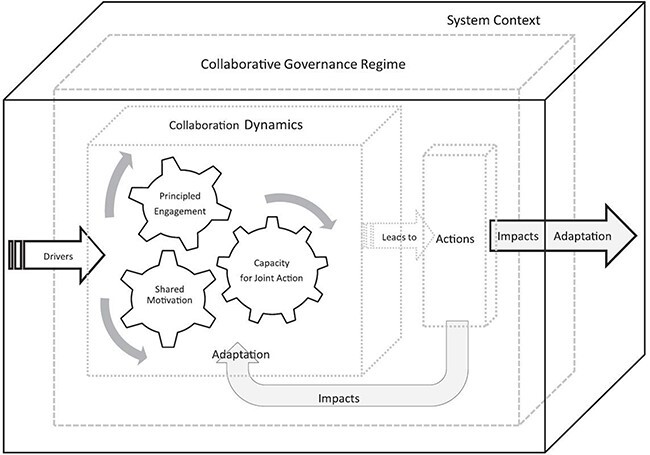
Emerson *et al*.’s integrative framework for collaborative governance (Emerson *et al*., 2012)

We used Emerson *et al*.’s integrative framework for collaborative governance, consisting of three nested dimensions—the general system context, the collaborative governance regime and its dynamics and actions—to explore how collaborative dynamics influence shared motivation in the tobacco control policy process (Emerson *et al*., 2012). The collaborative dynamics dimension contains the interactive components of principled engagement, shared motivation and capacity for a joint action. The consultative meetings constituted the ‘collaborative governance regime’ of interest, with a special focus on ‘shared motivation’ (Inner cycle: Figure 1). This concept consists of mutual trust and understanding, internal legitimacy and commitment. ‘Mutual trust’ happens over time as stakeholders work together and prove to each other that they are reasonable, predictable and dependable. ‘Mutual understanding’ is the ability to understand and respect others’ positions and interests even when one might not agree. ‘Internal legitimacy’, the confirmation that stakeholders in a collective endeavour are credible, with compatible and interdependent interests, legitimizes and motivates ongoing collaboration. ‘Shared commitment’ therefore enables stakeholders to cross organizational or sectoral boundaries and commit them to a shared collaborative vision. We have previously analysed principled engagement, another of the core concepts of collaboration dynamics, in tobacco control policy development in a separate article (Silumbwe *et al*., 2023).

The study framework is appropriate as it aligns with the complex and contentious nature of tobacco control policy development that requires the involvement of diverse stakeholders as applied elsewhere (Schneider *et al*., 2019). It allows us to pinpoint context-specific dynamics within the collaborative regime that shape tobacco control policy development. In this instance, focusing on shared motivation, we unpack obstacles to its realization and explore strategies to overcome these challenges.

### Study population, sampling and recruitment of study participants

The target population consisted of stakeholders from the public sector (Health, Agriculture, Trade and Commerce, Local Government and Housing, Justice, and Community Development and Social Welfare), civil society (Centre for Policy and Trade, the Non-communicable Diseases Alliance and the Tobacco Free Association of Zambia) and international organization (WHO) that attended the consultative meetings over the last 10 years. We purposively identified the participants by checking attendance registers, email lists and meeting minutes. Using snowball sampling, we consulted the initial group of stakeholders for suggestions on who else to include that had participated in the tobacco control policy process. Furthermore, the MoH officials in the Department of Non-communicable Diseases and Mental Health Unit provided appropriate guidance on people to recruit for the study.

### Data collection tools and techniques

We conducted 27 key informant interviews with tobacco control stakeholders (Table 1). The principle of theoretical saturation, which stipulates that data collection be stopped when additional interviews do not provide any new insights, guided the number of interviews performed (Rowlands *et al*., 2016). The framing of the questions was steered by the integrative framework for collaborative governance (Emerson *et al*., 2012). Specifically, the interview guide included questions framed to elicit perspectives on the four elements of shared motivation: trust among tobacco control stakeholders, mutual understanding, internal legitimacy and shared commitment.

**Table 1.** Study participants

Sector	Number of interviews
Civil society Centre for Policy and Trade Non-communicable Diseases Alliance Tobacco Free Association of Zambia	4
WHO	2
Ministry of Justice	2
MoLG	2
Ministry of Labour	1
MoH	5
MoA	4
MoC	2
Ministry of Education	2
MoF	3
Total interviews	27

The interviews were conducted between May and September 2021, and potential participants were contacted either in person or via mobile. Each of the interviews lasted between 35 min and 1 h, and they were held at a location convenient to the respondents. For those who were unable to meet physically, interviews were also conducted using the online meeting platform Zoom. All interviews were conducted in English, audio recorded and transcribed verbatim.

### Data analysis

We applied thematic analysis using NVivo version 12 Pro Software to facilitate the sorting, organization and coding of data (Braun and Clarke, 2012). This analysis was partly deductive, in that we organized the results into the main thematic areas drawn from the study framework—trust, understanding, commitment and legitimacy. It was also inductive in that the sub-themes emerged through an iterative process of discussing and reclassification of codes between the primary author and the research team. Since the main themes were predefined, the first phase of the data analysis process commenced with the primary author familiarizing himself with the data by reading the first five transcripts, identifying initial sub-themes and discussing them with the research team. Once the sub-themes were agreed upon, they were categorized according to the appropriate components of shared motivation. In the second phase, a coding structure comprising thematic definitions was developed and imported to NVivo for coding the rest of the transcripts. After every coding activity, where new sub-themes were identified, the team discussed and made appropriate modifications.

## Results

The results are organized according to the key elements of shared motivation in the study framework, which consist of four main themes with two to three sub-themes each (Table 2).

**Table 2.** Main and emerging themes

Main themes	Sub-themes
Factors shaping mutual trust	Concerns about associated loyalties
	Fear of ban of the tobacco production
	Silo-mentality and lack of comprehensive dialogue
Factors influencing mutual understanding	Limited information sharing
	Lack of reliable local evidence to influence stakeholder engagements
Factors shaping internal legitimacy	Sector representatives lack authority to make critical decisions
	Perceived lack of contextualization of proposed policy content
Factors affecting shared commitment	Acknowledgement of tobacco control multisectoral action
	Lack of political will from other sectors
	Limited allocation of funds to tobacco control by government

### Factors shaping mutual trust

#### Stakeholder concerns about associated loyalties

The civil society and health sector stakeholders expressed that politicians and other sectors such as agriculture and finance could not be trusted to act on the tobacco control policy because they were compromised financially by the tobacco companies. As one CSO leader recounted:

*The tobacco industries are way ahead of us. They talk to some of the stakeholders, offer them big money. You find that amongst the people that are supposed to participate in the tobacco control policy processes, they are being compromised; hence, they derail the policy* (CSO).

Another issue affecting trust among the stakeholders according to the agriculture sector was the perception that the health sector stakeholders were concerned with meeting the objectives and pleasing international partners, as opposed to paying attention to the local contextual realities of tobacco control:

*We should be mindful not to do things to please funders. Some people behave that way. Instead of being level-headed and principled enough to look at the interest of the country, people, and their economy, they tend to be influenced by outsiders* (MoA).

On the contrary, the health sector stakeholders revealed that strategic information, including proposed policy recommendations from the collaborative meetings, had been leaked to the tobacco companies, contributing to an environment of lack of trust. One representative of the MoH expressed it in this way:

*It is important to know who supports the tobacco industry or not in these meetings because we share information. Our colleagues are swift. They share certain information you wouldn’t want the industry to have. In our midst are stakeholders who are double tongued. They go to industry and come to tobacco control* (MoH).

#### Fear of a ban on tobacco production

Another factor that affected trust among the stakeholders was the fear that the proposed policy would enforce an outright ban of tobacco. In particular, the agriculture sector expressed fear that even if the proposed tobacco control policy may not explicitly state it, this might ultimately turn out to be the case with time. This was expressed by one of the officials at the Ministry of Agriculture (MoA):

*Let me not sugar-coat what might be sugar-coated in the bill* [policy] *that is advocating for what might be an outright ban. It’s the argument; that’s my fear! It might explode into an outright ban. Some of the provisions might not be friendly to the tobacco players. It’s just that, that’s my fear of the provisions* (MoA).

On the contrary, the health sector stakeholders felt that the fear was misplaced. They were clear that the policy would only regulate tobacco consumption and that the ban was a rumour perpetuated by tobacco companies:

*We are not saying we are going to ban tobacco production, but instead the use of tobacco should be controlled. That’s where now certain people from other sectors, particularly with the facilitation of the industry, paint a picture that once passed, the policy will be no more tobacco business* (MoH).

#### Silo-mentality and lack of comprehensive dialogue

Most stakeholders indicated that one of the factors impeding trust among them was the lack of comprehensive dialogue regarding the tobacco control measures in Zambia. The participants from the Ministry of Commerce (MoC) explained that most of the stakeholders operated in silos regarding tobacco control, which affected trust among them:

*We are an isolated people. There is a person here who seems to understand the tobacco issue in perspective. I think that is not enough. We should be striving to have all the strategists and policymakers understand the public health side as well as the business and revenue sides of it* (MoC).

In this regard, several stakeholders suggested different approaches for building trust among them, including exercising patience with one another, and being flexible and honest when discussing tobacco control matters during the consultative meetings:

*The Ministry of Health should not go with predetermined solutions. I think they should go into these meetings with an open mind to be able to learn and influence other stakeholders from other government departments to understand why it’s important for the Zambian people and its economy to regulate tobacco* (MoA).

### Factors influencing mutual understanding

#### Limited information sharing

All participants agreed that there was a need to improve information sharing regarding tobacco control efforts across sectors to enhance mutual understanding. For example, those from the Ministry of Finance (MoF) argued that they had been consistently raising taxes on tobacco over the years. However, this information was unknown to the other stakeholders. Further, health sector stakeholders added that there was a need to share information on tobacco use with policymakers such as parliamentarians to enable them to understand the urgency of the policy:

*We think that the most important thing to do is information sharing. We need to get the information to the right people at the right time, and the right time for this policy is now. We need to learn from one another regarding tobacco issues and possible interventions* (CSO).

#### Lack of reliable local evidence to influence stakeholder engagement

The stakeholders from the health and civil society sectors explained that the lack of nationally representative local evidence on tobacco use contributed to the privation of mutual understanding regarding the value of a comprehensive tobacco control policy in Zambia. One participant from the civil society described how the lack of compelling local evidence propagated misunderstanding among tobacco control stakeholders:

*Sometimes they were misunderstanding that the tobacco control fight is insincere; it’s not what it is. For example, if you’re going to talk about tobacco control in the country, give us the real evidence [data], and be clear on how big the problem is. Sometimes the health argument was not clearly understood by other stakeholders* (CSO).

In addition, sectors such as finance, agriculture and commerce questioned the credibility of the evidence used by the MoH to show the magnitude of the tobacco problem in Zambia. They argued that such evidence did not reflect local realities regarding tobacco smoking, which further compounded misunderstanding among stakeholders. For example, one participant from the agriculture sector commented:

*The Ministry of Health provides data, and people wonder where they got it from. They provide alarming data that seem not to align with the realities on the ground. The other ministries argue that this is not true because it’s not in the government statistics department, which is the arm doing research on behalf of government* (MoA).

### Factors affecting internal legitimacy

#### Sector representatives lack authority to make critical decisions

The stakeholders from the health sector indicated that the legitimacy of the deliberations in the consultative meetings was affected by sectors assigning representatives with limited authority to influence decisions on tobacco control in their respective institutions:

*Senior people are invited to the meetings, but in most cases those of lower rank are the ones sent to attend. If such a person has no influence, they can’t bring any change*, [it does not matter if you] *bring good ideas. If the focal point person does not even have the courage to approach his head of department or director, then it’s going nowhere* (MoH).

To address the above challenge, the health sector stakeholders had proposed, though without success, that higher-ranked officials should be invited to enhance the legitimacy of the decisions made in the meetings:

*We requested that the people who are invited be at the level of assistant director or director at best for them to come to the meeting. So that when we decide, It’s a meaningful decision. We can argue there and then and agree*(MoH).

#### Perceived lack of contextualization of proposed tobacco control policy content

The stakeholders from commerce, finance and local government were critical of some of the proposed tobacco control policy recommendations, as they perceived them to be unrealistic to the Zambian setting, which affected the legitimacy of the policy content. They emphasized the need for the tobacco control policy to adapt some of the interventions suggested in the WHO FCTC to the local context, and not reproduce it as is, to ensure local relevance, as one local government official put it:

*We cannot just handpick whatever recommendations from the Western world in the FCTC. It’s important that we understand what is happening in our country. Because here, of course, when you look at the population and the smoking habits, yes, we can say there are number of people who smoke, but you cannot compare to some developed countries* (MoLG).

For example, there were concerns about some tobacco control policy recommendations, such as having a predetermined tax on cigarettes. The stakeholders from the MoF were against such prescriptions because they believed that the MoH, which is the custodian of the tobacco control policy, was not privy to the considerations required to arrive at a particular tax policy rate for any product:

*There was one provision in the draft policy that suggested that the Ministry of Health shall prescribe the excise duty on tobacco. We were against this. We suggested that they use “may” instead of “shall” so that they give the finance a leeway to make their own decision. We made our submissions when the draft was circulated, but this was not addressed* (MoF).

### Factors affecting shared commitment

#### Acknowledgement of tobacco control multisectoral action

Most of the stakeholders acknowledged that the nature of the tobacco control problem required multiple actors, and getting their commitment was critical for successful policy-making and implementation, as one MoH official aptly explained:

*The nature of the tobacco problem needs government commitment. Leadership is mandatory because even after you have enacted the policy, if there is no coordination, it will be difficult because various stakeholders have to participate. That’s why in the whole process, you must do a lot of consultations. It’s an uphill battle, and that’s why this policy has been delayed* (MoH).

#### Lack of political will from other sectors

Some stakeholders felt that there was a lack of political will from certain sectors of the government to address tobacco use. Several echoed a lack of clarity regarding Zambia’s position on the tobacco control policy, which is notable in the contradictions among government sectors. According to the civil society stakeholders, this lack of clarity provided space for the tobacco industry to influence the policy process, as one of their representatives described:

*When we engage with ministries, the interests of the different ministries take charge. You find a little bit of hesitancy in some of the ministries to engage in certain tobacco control activities. That’s where you notice the influence of external forces*[tobacco industry] *because if the ministries cannot agree and have different positions, it becomes easy for them to influence the process* (CSO).

The stakeholders from the health sector explained that, in most instances, the tobacco consultative meetings would agree on issues to include in the draft policy, which were later rejected at the ministerial level. One MoH official had this to say:

*The problem is that if we agree in these consultative meetings, we believe we’ve made a commitment. But when another decision is made by respective ministries, we have no control as Ministry of Health, because we sat down and agreed, and when they go away and say this is not what they*[other sectors] *want, as the Ministry we have no control* (MoH).

In the same line, a civil society representative described how the MoH supported the tobacco control policy process but contended with the lack of political will from other ministers in the cabinet:

*I must confess that we have had it before, even with the previous government. Our Minister of Health then was in full support, but he had rebuttals from his other cabinet colleagues from the line ministries* (CSO).

#### Limited allocation of local funds to tobacco control

The stakeholders from the health sector and civil society recounted that there was scarce allocation of funds to the tobacco control policy process by the government. They explained that all financial resources availed for the tobacco control policy development meetings were provided by the civil society and international organizations, which showed the lack of government commitment. In addition, limited local funding entailed a lack of collective voices to hold the government accountable, as one civil society leader explained:

*We are coordinating ourselves, but as I mentioned, the resources to hold meetings are so often not there; that is what is lacking. Because even for us to have a formidable voice against tobacco, we need to have resources that will make us work hard and hold government accountable. Unfortunately, government commits little to no resources to the process, except for funding partners* (CSO).

## Discussion

We aimed to explore how the collaborative dynamics within the tobacco control policy process shaped shared motivation among stakeholders in Zambia. This study makes important contributions to how we understand tobacco control policy development in LMICs. Firstly, while the tobacco industry plays an influential role in derailing tobacco control policies, the dynamics within the policy formulation process may impact how long it takes to deliver the policy. Secondly, we highlight the importance of a critical component in policy development, namely ‘shared motivation’, which is often overlooked but is vital for both the success of policy development and implementation efforts. Thirdly, a crucial aspect in unlocking the often-contentious tobacco policy process involves understanding context-specific collaborative dynamics associated with certain perceptions and practices that shape shared motivation among stakeholders. Our findings align closely with similar studies on the dynamics of intersectoral collaboration within the tobacco control policy process (Rosser, 2015; Kusi-Ampofo, 2021; Mondal *et al*., 2021). In the following, we discuss these findings in relation to agenda setting and engagement, stakeholder perceptions of the proposed policy content, the power dynamics and their impact on the different dimensions of shared motivation.

### Agenda setting and stakeholder engagement

There was broad acknowledgement of a need for tobacco control intersectoral action among the stakeholders. Such recognition has been reported to facilitate development of the tobacco control policy in Kenya and South Africa, where the inadequacies of the then policy framework were aptly acknowledged by stakeholders to initiate a collaborative policy process (Mohamed *et al*., 2018; Sanni *et al*., 2018). Moreover, this recognition of intersectoral efforts may contribute to elevation of the tobacco control policy onto the agenda-setting stage, where issues are given priority in the policy-making process. However, like others, our study showed that agenda setting remains a complex undertaking shaped by several factors, including limited trust and understanding, uncertainty about the tobacco industry and conflicting mandates among government departments (Kumar *et al*., 2022; Silumbwe *et al*., 2023). These dynamics significantly affect engagement beyond the health sector.

Limited sharing of information undermined trust and understanding during stakeholder engagement. Several studies underscore the importance of trust in facilitating the alignment of individual interests with those of others (Ahern and Hendryx, 2003; Gilson, 2003; Oh and Bush, 2016). Trust is fundamental for negotiating relationships and enabling effective communication in policy undertakings. Our findings suggest that engagement in the tobacco control policy development was constrained by limited understanding of sector responsibilities in tobacco control efforts, resulting from inadequate information sharing. Similarly, our recent study found that tobacco control information sharing in Zambia was less frequent outside of the policy consultative meetings, and mostly restricted to the MoH and related CSOs (Silumbwe *et al*., 2023). This weak sharing of information affects trust by perpetuating negative sentiments against the tobacco control policy efforts (Barry *et al*., 2022).

The scarcity of reliable local evidence on tobacco smoking undermined trust during stakeholder engagement. Evidence enables contextually relevant decision-making that enhances trust in the policy process (Riege and Lindsay, 2006; Malama *et al*., 2021). Moreover, evidence is essential to counter deceptive economic arguments relating to the country’s development often used by the tobacco industry (Matthes *et al*., 2020). However, contradictions regarding the credibility of available evidence on the magnitude of the tobacco problem in Zambia negatively affect evidence use in tobacco control policy-making. This finding suggests the potential role of population representative data in highlighting tobacco harms, at-risk populations, social determinants and trends in tobacco use. Specifically, data from the STEPS survey and the Investment Case have a greater role in informing arguments for the tobacco control policy (Government of the Republic of Zambia, MOH, 2019; Pengpid and Peltzer, 2020; Silumbwe *et al*., 2022). Furthermore, a situation analysis of the tobacco burden, normally a requirement for government policy, which is supported by all sectors, may help to improve evidence use and engagement (Zulu *et al*., 2022).

### The policy content and process legitimacy

The findings show that part of the reasons the tobacco control policy has dragged is the fear that it will ban production/manufacturing and a perceived lack of contextualization of the proposed content. While the MoH argues that this fear is misplaced and perpetuated by the tobacco industry, our findings suggest that there is a need for clarity regarding alternative livelihoods for tobacco farmers, to enhance trust in the policy process and its content. According to a recent study, Zambian farmers engage in tobacco cultivation for various reasons (Appau *et al*., 2020). Alternative livelihoods interventions in the tobacco control policy dialogue should not take a one-size-fits-all approach but consider tobacco-producing districts’ unique features (Lencucha *et al*., 2022). Furthermore, the perception that the policy content is contextually relevant is critical for the support of stakeholders (Simooya *et al*., 2023). However, our findings show that as long as stakeholders perceive that the proposed policy content does not address local realities, it will be a challenge to develop a tobacco control policy. Moreover, stakeholder reservations regarding some of the proposed policy content affects its legitimacy—the normative approval by stakeholders, which increases compliance with, and raises satisfaction with the policy process (Mazepus, 2018). The legitimacy of a policy rests on it being seen as reflecting the aspirations and content of all those involved in its development (Tyler, 1997; Rothstein, 2012; Mazepus, 2018).

### Power dynamics and commitment

Both overt and covert exercises of power, including the lack of government allocation of funds to tobacco control and the assignment of sector representatives with limited authority, were noted, respectively. Limited local funding not only reinforces stakeholder suspicions over associated loyalties but also entails that essential policy activities may not occur without external support, showing a lack of government ownership and commitment. Moreover, junior representatives may lack commitment to implement decisions taken in the meetings within their respective sectors, knowing that they can always be overruled by higher authorities. The impact of covert power exerted by the industry on government is evident in the inertia to support the tobacco control policy within certain sectors. According to our recent study, some stakeholders see no need for a comprehensive tobacco control policy but rather argue that the country should focus on strengthening existing subsidiary tobacco laws (Silumbwe *et al*., 2023). These stakeholders often echo familiar claims made by the industry, implying its deep-rooted influence on the policy process (Barry *et al*., 2022). Additionally, overt and covert tactics, such as extensive product marketing, maintaining low product prices, systematically violating tobacco control legislation and galvanizing political support, are often used by the industry to sway policy direction across LMICs (Gilmore *et al*., 2015; Astuti *et al*., 2020; Matthes *et al*., 2021). Our findings offer valuable lessons for implementing Article 5.3 of the WHO FCTC, emphasizing the need for governments to protect the tobacco control policy process from vested interests of the industry (Barry *et al*., 2022). It is imperative to adopt measures that limit and ensure transparency in the interactions with the industry while avoiding conflicts of interest among stakeholders in LMICs.

To the best of our knowledge, this is the first time the integrative framework for collaborative governance has been used to study tobacco control policy-making (Emerson *et al*., 2012). Its broad applicability allows for the analysis of collaborative regimes, such as the tobacco control policy process, and its inherent dynamics across diverse contexts (Emerson and Nabatchi, 2015a; 2015b). However, while the framework provides valuable insights into understanding the collaborative dynamics, making a definitive claim that addressing them will lead to the enactment of the tobacco control policy might be overly deterministic. Nevertheless, the lessons learnt from unpacking these dynamics that shape shared motivation hold relevance in similar contexts seeking to overcome prolonged challenges of contentious tobacco control policy-making in LMICs.

### Strengths and limitations

The strengths of this study include the collection of data from multiple stakeholders who have been actively engaged in the tobacco control policy process, which not only provides richness in perspectives but also enables triangulation of perspectives across various sector representatives. The reflective and iterative nature of the data analysis, whereby the codes and themes were identified, discussed by the whole research team and then collectively modified and agreed upon to ensure that they addressed the study question, increases the validity and trustworthiness of our findings (Gunawan, 2015; Fitzpatrick, 2019). Furthermore, the use of the integrative framework for collaborative governance adds an insightful dimension to understanding Zambia’s tobacco control policy process using a governance lens. Nevertheless, there are also some limitations to be considered. Firstly, the process of health policy-making is not overt or clearly bounded—i.e. ways in which policy decisions ‘emerge’ and are often unobservable to the researcher can be particularly difficult to unpack and explain accurately. Secondly, the depth of the data collected may be limited due to the challenge of conducting interviews with policymakers who might not recall all details relating to the policy process. Lastly, purposive selection of study participants may have introduced some inherent biases that have potential to influence the study findings.

## Conclusion

In conclusion, we note a limited sense of shared motivation among the tobacco control stakeholders in Zambia. Our study has uncovered collaborative dynamics that exacerbate a lack of trust, understanding, legitimacy and commitment among tobacco control stakeholders, from which lessons can be drawn and inferred to similar settings in LMICs. These dynamics have a detrimental effect on engagement in collaborative efforts aimed at developing a mutually beneficial tobacco control policy. To foster trust, it will be essential to adopt strategies within the tobacco control policy processes that create opportunities for open discourse and respectful interactions among stakeholders, thereby reducing tensions, fear and suspicion. Improving understanding among tobacco control policy stakeholders will require promoting a cultural shift towards collaborative information sharing. Furthermore, to enhance legitimacy and commitment, it will be important to ensure that tobacco control policy stakeholders have the requisite power and authority to influence appropriate policy actions in their respective sectors. Lastly, we recommend that future research is embedded and builds further on understanding collaborative dynamics within similar health policy processes and what strategies can be adopted to expedite the development of highly contested health policies. This shows the importance of shared motivation for health policy processes where there are conflicting interests.

## Data Availability

The data underlying this article are available in the article and in its online supplementary material.

## Abbreviations

CSO = civil society organization

MoA = Ministry of Agriculture

MoC = Ministry of Commerce

MoH = Ministry of Health

MoLG = Ministry of Local Government

MoF = Ministry of Finance

LMICs = low- and middle-income countries

WHO FCTC = World Health Organization Framework Convention on Tobacco Control.

## Reflexivity statement

The authorship team consists of three senior members and one junior researcher. Two researchers originate from the global north, while three hail from the global south. Among the researchers, two predominantly employ quantitative methods, one utilizes mixed methods and the remaining two are experienced qualitative researchers who have conducted similar work in the past. Furthermore, three of the researchers primarily specialize in epidemiology, whereas the other two focus on health policy and systems research. This multidisciplinary team brought diverse expertise and perspectives to this research project.
